# P002 Paget disease of bone in routine practice: three cases demonstrating distinct presentations and complications

**DOI:** 10.1093/rap/rkag113.032

**Published:** 2026-11-03

**Authors:** Lih Yin Chong, Anupama Nandagudi

**Affiliations:** Basildon University Hospital, Basildon, United Kingdom; Basildon University Hospital, Basildon, United Kingdom; Anglia Ruskin University, Cambridge, United Kingdom

## Abstract

**Introduction:**

Paget’s disease is a disorder of abnormal bone remodelling and is often underdiagnosed, especially when asymptomatic. It is often detected via raised alkaline phosphatase (ALP). Misattribution to liver disease can often lead to delay in diagnosis of Paget’s disease, which can lead to complications such as bone deformities, hearing loss and increased risk of fractures. This case series presents three patients with Paget’s disease demonstrating varied clinical presentations, anatomical distributions, and complications encountered in routine clinical practice.

**Case description:**

We present a series of three patients diagnosed with Paget disease of bone with differing presentations and disease patterns.

**Case 1** involved a 77-year-old British White male who presented with left knee pain associated with isolated elevation of ALP. Initial biochemical investigation demonstrated a markedly elevated ALP level of 1550 U/L with adjusted calcium of 2.45 mmol/L. Bone scintigraphy demonstrated extensive polyostotic involvement affecting the skull, lower thoracic and lumbar spine, pelvic bones, and proximal to mid left femur, with associated femoral deformity. The patient subsequently developed deafness as a disease-related complication. Treatment with intravenous pamidronate was initiated. Follow-up demonstrated biochemical response with ALP improving to 82 U/L. No family history of Paget disease was identified.

**Case 2** involved a 65-year-old British White female who presented with swelling affecting the left side of the mouth. ALP at diagnosis was elevated at 369 U/L with normal adjusted calcium of 2.33 mmol/L. Bone scintigraphy identified isolated jaw involvement. The disease course was complicated by chronic jaw infection with persistent purulent discharge. The patient received zoledronic acid treatment. Latest ALP improved to 246 U/L. There was no relevant family history.

**Case 3** involved a 68-year-old Black Caribbean male presenting with multiple joint pain and incidentally raised ALP. Initial ALP was 340 U/L with adjusted calcium of 2.41 mmol/L. Bone scintigraphy and SPECT/CT demonstrated skull involvement. Disease progression was associated with deafness. Treatment with zoledronic acid was administered, with subsequent reduction in ALP to 190 U/L. Family history was negative.

**Discussion:**

• This case series highlights the heterogeneity of Paget disease in terms of presentation, anatomical involvement, and complications.

• Despite sharing the same diagnosis, the three patients presented with:

• Localised musculoskeletal pain and marked biochemical abnormality (Case 1)

• Craniofacial swelling with jaw involvement (Case 2)

• Non-specific joint pain with incidental biochemical detection (Case 3)

• Role of ALP:

• Elevated ALP was the key diagnostic trigger in all cases.

• Normal calcium levels emphasise that Paget disease may present with isolated ALP elevation.

• Role of imaging:

• Bone scintigraphy was essential for defining disease extent.

• Case 1 demonstrated extensive polyostotic disease with deformity.

• Cases 2 and 3 showed focal craniofacial involvement.

• Imaging beyond the symptomatic region is important because clinically silent lesions may coexist.

• Complications:

• Deafness occurred in two patients with skull involvement, likely due to cochlear or neural compression.

• Chronic jaw infection in Case 2 illustrates the morbidity associated with craniofacial disease.

• Treatment response:

• All patients received intravenous bisphosphonates (pamidronate or zoledronic acid).

• All demonstrated biochemical improvement with reduction in ALP.

• Biochemical response does not always correlate with symptom resolution, highlighting the need for clinical follow-up.

• Clinical implications:

• Clinicians should consider Paget disease in older adults with unexplained ALP elevation.

• Early detection may reduce long-term complications, though the benefit of treating asymptomatic disease remains an area for discussion.

**Key learning points:**

• Paget disease of bone has variable presentations, ranging from focal bone pain to craniofacial symptoms or incidental biochemical abnormalities.

• A high index of suspicion is required in older adults with unexplained ALP elevation.

• ALP is a sensitive initial marker; normal calcium does not exclude Paget disease.

• Bone scintigraphy is valuable for assessing disease extent and detecting clinically silent lesions.

• Anatomical distribution influences prognosis:

• Skull involvement may lead to hearing loss.

• Jaw involvement may predispose to chronic infection and local morbidity.

• Bisphosphonates remain the mainstay of therapy, with biochemical improvement seen in all cases.

• Clinical monitoring is essential because biochemical response does not always reflect symptom burden.

• Early recognition and treatment may help reduce complications such as deformity, osteoarthritis, and fracture risk.

• Family history is often absent, and disease may occur sporadically.

• This case series reinforces the need for systematic evaluation of elevated ALP and awareness of atypical presentations.

**P002 Table 1:** 

	Case 1	Case 2	Case 3
Presentation	Musculoskeletal pain and high ALP	Craniofacial swelling with jaw involvement	Non-specific joint pain with high ALP
ALP level at diagnosis	1550	369	340
Location	Skull, lower thoracic and lumbar spine, pelvic bones, and proximal to mid left femur	Isolated jaw involvement.	Isolated skull involvement.
Complication	Deafness	Chronic jaw infection	Deafness
Family history	No	No	No

